# Resistant Hypertension Secondary to Severe Renal Artery Stenosis With Negative Duplex Ultrasound: A Brief Review of Different Diagnostic Modalities

**DOI:** 10.1177/2324709620914793

**Published:** 2020-03-22

**Authors:** Navya Vipparla, Asim Kichloo, Michael Stanley Albosta, Michael Aljadah, Farah Wani, Nazir Lone

**Affiliations:** 1 St. Mary’s of Saginaw Hospital, Saginaw, MI, USA; 2 Central Michigan University, Saginaw, MI, USA; 3 Bassett Medical Center, Cooperstown, NY, USA

**Keywords:** renal artery stenosis, hypertension

## Abstract

Renal artery stenosis is a cause of resistant hypertension, which can present with several features such as severe hypertension, deterioration of renal function (with or without associated angiotensin-converting inhibitor or angiotensin receptor blocker therapy), and flash pulmonary edema. When evaluating for the presence of renal artery stenosis, the most widely utilized imaging modalities are duplex ultrasonography and computed tomography angiography. In this article, we discuss the case of a 77-year-old female who presented with shortness of breath and mild pulmonary edema, secondary to hypertensive emergency. Later, she was diagnosed with renal artery stenosis and underwent stent placement in the left renal artery. Our case highlights the different diagnostic modalities and emphasizes that the most commonly used screening, which is duplex ultrasonography, was performed on our patient but gave a false-negative result, despite high-grade stenosis, which was later diagnosed on computed tomography angiography.

## Case Report

A 77-year-old Caucasian female with a past medical history of carotid artery stenosis, hypertension, hyperlipidemia, and hypothyroidism presented to the emergency department with sudden-onset shortness of breath for a few hours with associated symptoms of orthopnea and nausea. She reported that her blood pressure had been difficult to control for the past 4 years with medications. Her vitals on presentation showed hypertension with a blood pressure of 250/96 mm Hg with a normal heart rate of 91 beats per minute. Pertinent physical examination findings included a carotid bruit on the right side, an ejection systolic murmur over the aorta area, and inspiratory crackles at bilateral bases on auscultation. An abdominal examination revealed faint abdominal bruits bilaterally. Positive laboratory findings included a BNP (brain natriuretic peptide) level of 384 pg/mL (range = 0-150 pg/mL), sinus tachycardia on electrocardiogram shortly after presentation with a heart rate of 102 beats per minute, and bilateral vascular congestion on chest X-ray. The patient was started on a labetalol drip, 4 L of supplemental oxygen via nasal cannula, and was given 60 mg intravenous furosemide with marked improvement of symptoms.

**Figure 1.**
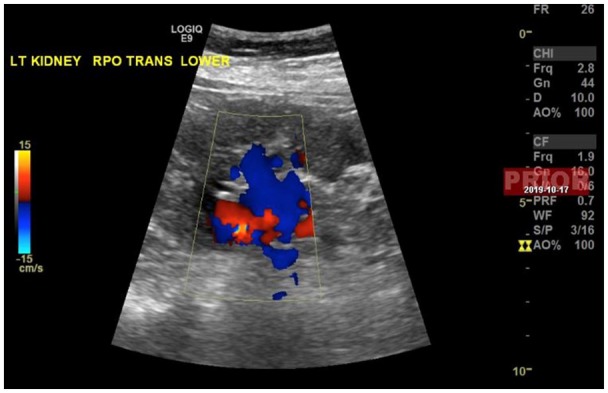
Duplex ultrasound of the left kidney showing patent blood flow to the kidney via the left renal artery.

**Figure 2.**
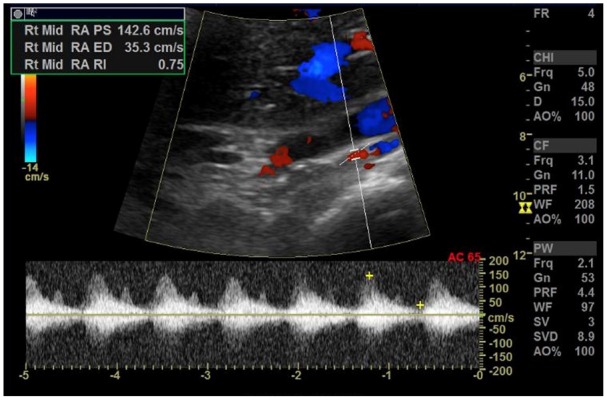
Duplex ultrasound of the right kidney showing patent blood flow to the kidney via the right renal artery.

Workup revealed an elevated renin level of 7.1 ng/mL/h (range = 0.5-4.0 ng/mL/h upright) and an elevated aldosterone level of 48.2 ng/dL (range = 4.0-31.0 ng/dL upright; Table 2). The patient was not hypokalemic (Table 1). The aldosterone levels were appropriately elevated for the elevation of renin implying secondary aldosteronism due to renovascular cause or dehydration. Furthermore, the lack of renin suppression led to a high suspicion for renal involvement. Renal ultrasound was unremarkable for any structural causes, and renal duplex ultrasound showed patent bilateral renal arteries (Figures 1 and 2). Subsequent autoimmune workup was negative, and the patient had metanephrine levels within normal ranges (Table 3). The patient continued to improve and was discharged home on clonidine, spironolactone, amlodipine, and isosorbide mononitrate for blood pressure control.

**Table 1.** Basic Metabolic Panel Values.

	Hospital Day 0	Hospital Day 2	Inpatient Discharge	One Week Later (Outpatient)	Post-Stent Discharge	Reference Range
Glucose	134	113	100	109	97	70-125 mg/dL
BUN	38	31	33	64	20	7-26 mg/dL
Creatinine	1.1	1.7	1.3	3.7	4.9	0.6-1.2 mg/dL
Calcium	10.1	8.1	9.5	10.5	8.7	8.5-10.7 mg/dL
Sodium	139	135	133	130	133	135-145 mmol/L
Potassium	3.8	3.8	4.2	5.2	3.6	3.5-5.0 mmol/L
Chloride	103	104	101	94	96	98-112 mmol/L
CO_2_	24	21	23	23	27	22-32 mmol/L
Anion gap	12	10	9	13	10	3-11 mmol/L
GFR	48	29	34	12	9	>60 mL/min/1.73 m^2^

Abbreviations: BUN, blood urea nitrogen; GFR, glomerular filtration rate.

**Table 2.** Aldosterone and Renin Values.

	Day 0	Reference Range
Aldosterone	48.2	4.9-31.9 ng/dL upright
Renin	7.1	0.5-4.0 ng/mL/h upright
ARR (angiotensin renin ratio)	6.7	ng/dL

**Table 3.** Metanephrine/Normetanephrine.

	Day 0	Reference
Metanephrine	112	39-143 µg/d
Normetanephrine	348	109-393 µg/d

**Figure 3.**
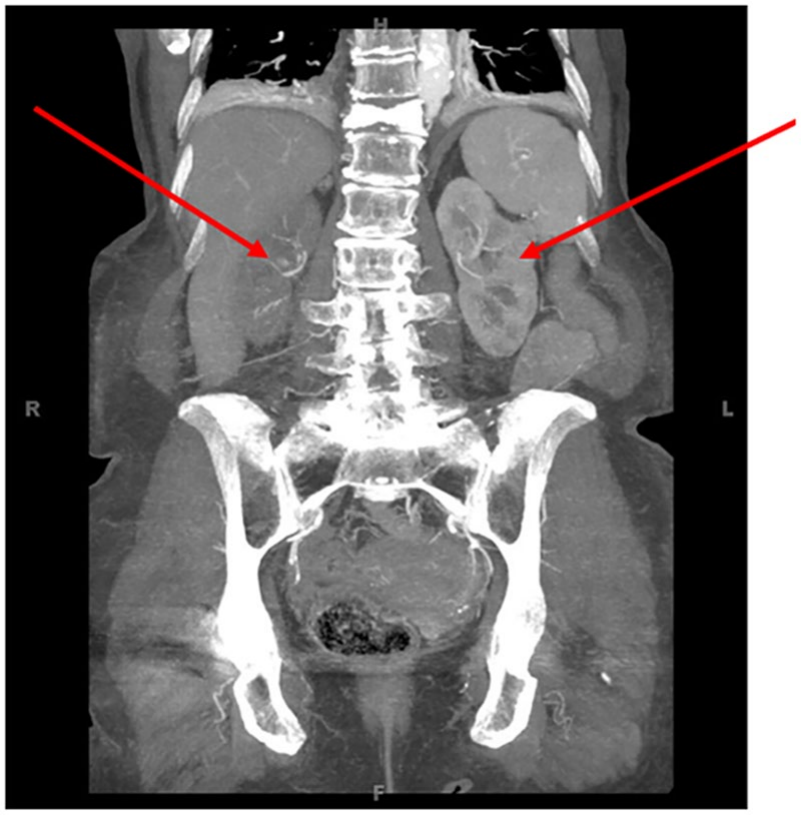
Computed tomography angiogram of the abdomen and pelvis showing a sharp decrease in attenuation of the right kidney due to complete obstruction of the right renal artery.

**Figure 4.**
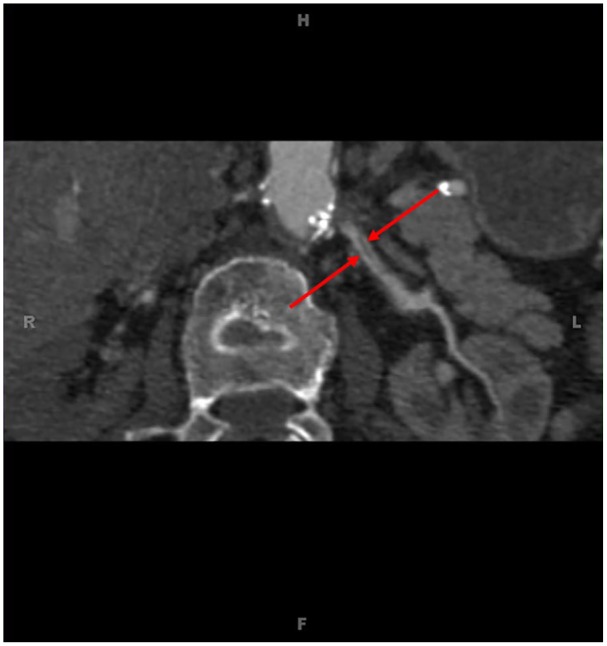
Computed tomography angiogram of the abdomen and pelvis and left kidney showing 2 renal arteries, a superior and inferior. It was determined that the inferior renal artery was 90% stenosed at the origin.

One week later, the patient received follow-up laboratory results as outpatient, which showed marked elevation of creatinine from 1.5 mg/dL on hospital discharge to 3.7 mg/dL. Spironolactone was discontinued by her primary care provider, as a result. On interviewing the patient, the primary care provider elicited that the patient has been using a nonsteroidal anti-inflammatory drug (NSAID) agent several times a day for migraine headaches, and this has been ongoing for several weeks. She was subsequently readmitted to the hospital for worsening kidney function, presumably an acute injury due to NSAID medications, acute tubular necrosis from accelerated hypertension, or renovascular hypertension as a differential diagnosis. She was started on intravenous fluids, and the blood pressure regimen was changed to avoid nephrotoxic medications; however, her kidney function continued to deteriorate. Temporary dialysis for oliguria and worsening kidney function was started, and a renal biopsy was done that showed cortical necrosis, a finding highly suspicious for renovascular origins despite prior negative duplex renal artery sonogram. A computed tomography angiogram (CTA) of the abdomen/pelvis with contrast was performed on her dialysis day, which showed complete occlusion of the right renal artery and 90% stenosis of left inferior renal artery (Figures 3 and 4). CTA was chosen over magnetic resonance angiography (MRA) for cost and ease of access purposes. Expert consultation subsequently determined that the right renal artery was not salvageable due to passing of the occlusion window, so the patient underwent immediate stent placement in the left renal artery. The patient’s blood pressure slowly started to stabilize and she was discharged on carvedilol 25 mg twice daily with good control. The patient’s creatinine levels stabilized at 4.0 mg/dL, and she continued to be dialysis dependent due to oliguria.

## Discussion

### Renal Artery Stenosis Typical Presentation

Patients with renal artery stenosis (RAS), which is defined as narrowing of the renal arteries, typically present with clinical characteristics of secondary hypertension. Characteristics from the patient history suggestive of RAS include hypertension that is severe, abrupt in onset, occurring at a young age, or resistant to treatment.^1^ Physical examination findings may include an abdominal bruit; severe retinopathy; occlusive vessel disease in other vasculature, such as carotid, coronary, or peripheral vasculature; and unexplained congestive heart failure or pulmonary edema.^2^ Laboratory results in patients with RAS may include elevated levels of renin, angiotensin, and aldosterone in addition to renal impairment (azotemia).^1^ Patients who have these clinical features should be evaluated further for the presence of RAS using imaging studies. The most common studies used to evaluate for the presence of stenosis include duplex ultrasonography (DUS), MRA, and CTA.^2^

### Fibromuscular Dysplasia Versus RAS

Secondary hypertension is defined as increased blood pressure due to an identifiable cause. Of all cases of hypertension, 5% to 10% are due to secondary causes, whereas 90% to 95% are primary (idiopathic) hypertension.^3^ Secondary hypertension should be considered in patients with abrupt onset or accelerating hypertension, malignant hypertension, excessive target organ damage for the level of blood pressure, new diastolic hypertension in patients >65 years old, resistant hypertension, and onset of hypertension before 30 years of age.^4^ One of the most common causes of secondary hypertension is renovascular hypertension, which can be further broken down into fibromuscular dysplasia and atherosclerotic RAS (ARAS). ARAS accounts for roughly 90% of cases of renovascular hypertension, usually diagnosed in patients older than 50 years of age with significant risk for atherosclerotic disease.^2^ The remaining 10% of cases are due to fibromuscular dysplasia, usually diagnosed between the ages of 25 and 50 years.^2^

Atherosclerotic renal artery stenosis typically leads to plaque development at the ostia of the renal artery as it branches from the abdominal aorta.^2^ ARAS is a progressive disease, with 51% of patients reporting worsening stenosis 5 years after diagnosis.^2^ Fibromuscular dysplasia is a common cause of hypertension in younger individuals, predominantly in females. It is a hyperplastic disorder that affects small- to medium-sized arteries. Arterial lesions in fibromuscular dysplasia tend to affect more distal portions of the renal artery, in contrast with the more proximal lesions consistent with ARAS.^2^ It does not often affect kidney function; however, it is associated with renal artery aneurysms and in some cases produces total occlusion of renal arteries.^2^

### Sensitivity and Specificity of Doppler Ultrasonography

When evaluating for the presence of RAS, one of the most widely utilized imaging modalities is DUS.^5^ The benefits of DUS include that it is noninvasive, radiation free, cost-effective, and is not contraindicated in patients with allergy to intravenous contrast or most importantly renal failure patients.^6^ It can provide an image of the renal artery, as well as a measurement of blood flow velocity and pressure waveforms. When considering its effectiveness as a screening tool, sensitivity ranges from 60% to 97% and specificity ranges from 85%to 99%.^5^ There are several drawbacks when using DUS for the diagnosis of RAS. These include low sensitivity, the fact that the success of this test is largely dependent on operator experience, and low image quality in patients with excess adipose tissue and bowel gas.^5^

Other common imaging modalities utilized in the diagnosis of RAS include CTA and MRA. The benefits of CTA include high sensitivity (90% to 98%) and specificity (85% to 94%)^5^; however, CTA is contraindicated in patients with renal failure and contrast allergy. CTA also exposes patients to radiation. MRA is the most accurate diagnostic test, with recent studies showing sensitivities from 90% to 100% and specificities from 88% to 100%.^5^ MRA is very accurate and can be used in patients with renal failure and contrast allergy; however, it is limited due to high cost and availability.

### Renal Artery Stenosis Diagnosis and Treatment

When patients present with several features, such as resistant hypertension, recent onset of severe hypertension, recent deterioration of renal function with or without associated angiotensin-converting inhibitor or angiotensin receptor blocker therapy, or flash pulmonary edema, an investigation for RAS should be initiated.^3^ The diagnosis should be made via imaging studies, due to the fact that laboratory evaluations often lack specificity.^7^ The imaging studies used most frequently are DUS, MRA, and CTA. DUS is often chosen because it is noninvasive, cost-effective, and can be used in renal failure patients.^6^ It is important to remember, however, that the sensitivity and specificity of this modality are the lowest among the 3 methods discussed. It is also important to remember that DUS is operator dependent and image quality may be limited in obese patients or those with excessive bowel gas.^5^ This is apparent in our case, in which an opportunity to diagnose a patient with signs and symptoms of RAS was missed based on a false-negative ultrasound. The diagnosis was later confirmed with a more sensitive and specific test, CTA.

Treatment of RAS revolves around the benefit of stenting versus medical therapy. Because decreased renal perfusion activates the renin-angiotensin-aldosterone system, an angiotensin-converting inhibitor or angiotensin receptor blocker is commonly used.^7^ Additionally, statins and antiplatelet therapy are included in RAS treatment due to the known benefits of these agents in patients with atherosclerotic disease. However, the ASTRAL trial looked to determine whether there was clinical benefit from revascularization when compared with medical therapy alone. At the conclusion of the study, it was determined that revascularization therapy was not associated with any benefit to renal function, blood pressure, renal or cardiovascular events, or overall mortality when compared with patients treated with antihypertensive, antiplatelet, and cholesterol-lowering medication alone.^8^ In fact, a number of patients in the trial who underwent revascularization suffered from serious complications due to the procedure, leading the authors to conclude that not only was there no significant benefit to revascularization, but it also carried significant risk.^8^

Revascularization is reserved for selected patients with severe RAS who are symptomatic and continue to have acute severe worsening of renal function. However, it is important to note that the authors of the ASTRAL trial noted a limitation that patients with severe RAS (those presenting with kidney injury or pulmonary edema) were unlikely to be included in the trial, as they likely were treated with revascularization immediately.^8^

## Summary

Our case was unique due to the fact that the most commonly used screening test was performed on our patient, giving us a false-negative result. This can be avoided by using an imaging modality with a higher sensitivity and specificity for detecting RAS, if the clinical suspension is high. Based on this case, it may be appropriate to recommend screening with CTA in patients with adequate renal function and without contrast allergy. Although these patients will be exposed to additional radiation, the ability of this modality to correctly identify patients with RAS will ultimately lead to less invasive and less costly testing, as well as more efficient treatment and better clinical outcomes.
